# Supplementary material for: Multiple interactions mediate the localization of BLTP2 at ER-PM contacts to control plasma membrane dynamics
Source: bioRxiv. 2025 Feb 8:2025.02.07.637094. Preprint. [Version 1] doi: 10.1101/2025.02.07.637094 (PMC11839039; doi:10.1101/2025.02.07.637094)
Supplement: Supplement 1 [file NIHPP2025.02.07.637094v1-supplement-1.pdf]

bioRxiv preprint doi: https://doi.org/10.1101/2025.02.07.637094; this version posted February 8, 2025. The copyright holder for this preprint (which was not certified by peer review) is the author/funder, who has granted bioRxiv a license to display the preprint in perpetuity. It is made available under aCC-BY 4.0 International license.

Supplemental Figure 1

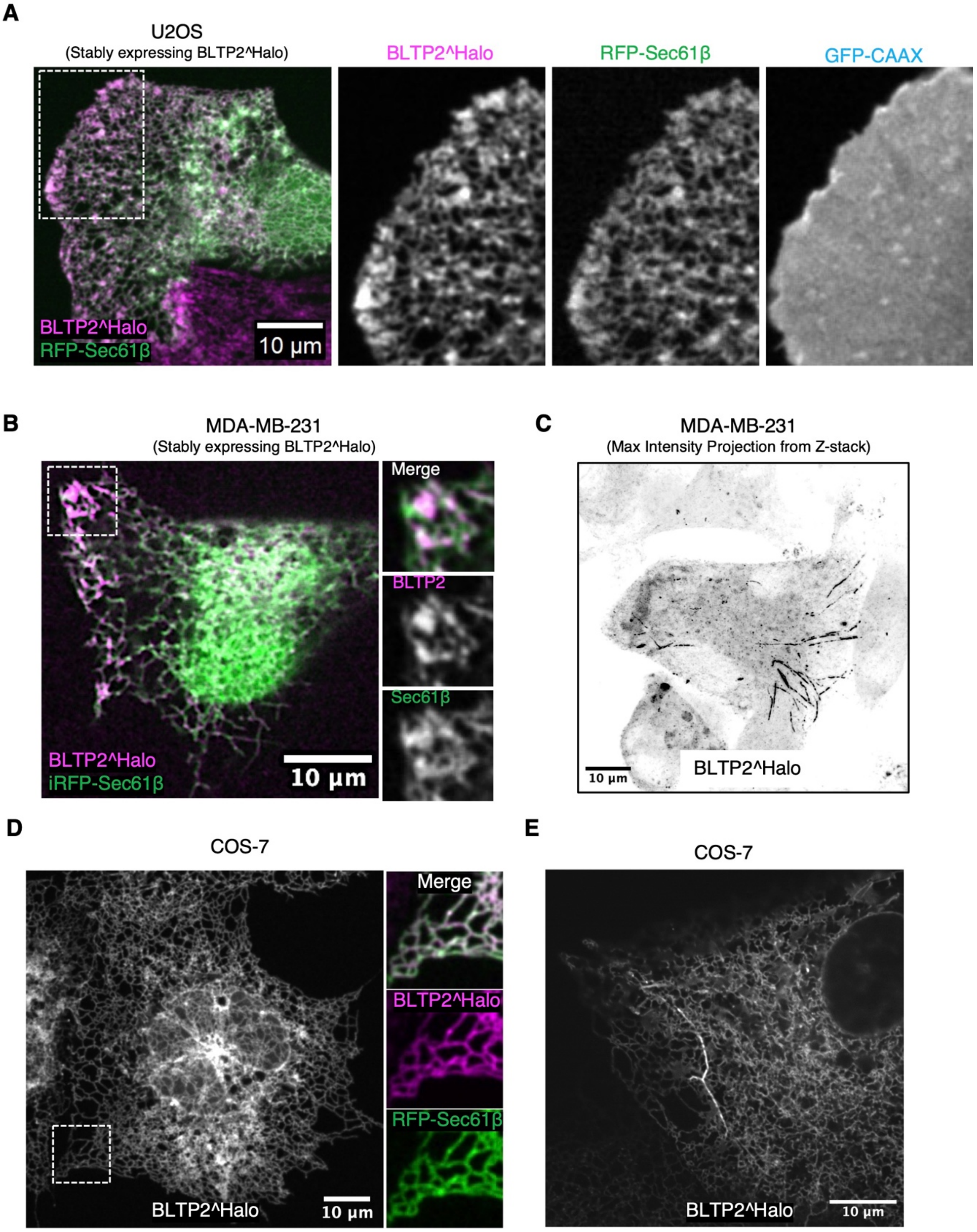

bioRxiv preprint doi: https://doi.org/10.1101/2025.02.07.637094; this version posted February 8, 2025. The copyright holder for this preprint (which was not certified by peer review) is the author/funder, who has granted bioRxiv a license to display the preprint in perpetuity. It is made available under aCC-BY 4.0 International license.

Figure S1. BLTP2^Halo show heterogeneous localization in different cell types

(A and B) U2OS (A) or MDA-MB-231 (B) cells stably expressing BLTP2^Halo show its localization throughout the ER (labeled with ER marker Sec61β) with an enrichment at ER-PM contacts.

(C) In some MDA-MB-231 cells, BLTP2^Halo show an enrichment on tubular structures similar to those observed in HeLaM cells.

(D and E) BLTP2^Halo mainly localizes throughout the ER in COS-7, as shown by colocalization with the ER marker RFP-Sec61β (D), and only occasionally it is enriched at some tubular structures (E).

bioRxiv preprint doi: https://doi.org/10.1101/2025.02.07.637094; this version posted February 8, 2025. The copyright holder for this preprint (which was not certified by peer review) is the author/funder, who has granted bioRxiv a license to display the preprint in perpetuity. It is made available under aCC-BY 4.0 International license.

Supplemental Figure 2

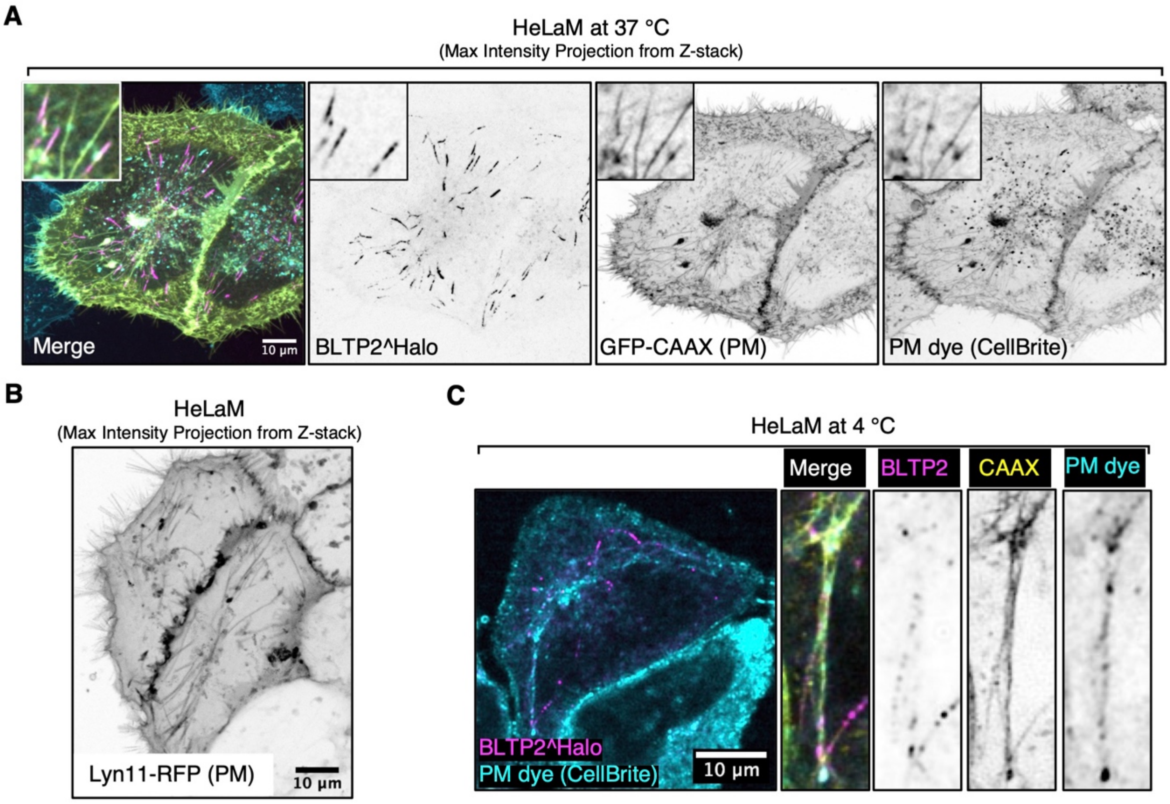

Figure S2. BLTP2-positive tubular endosomes are positive for PM markers

(A) BLTP2^Halo is enriched at tubular structures positive for the PM marker GFP-CAAX and for an extracellular membrane impermeable dye CellBrite labeled at 37 °C.

(B) The tubular structures of HeLaM cells are also positive for another PM marker, Lyn11-RFP.

(C) BLTP2-positive tubules are also labeled by CellBrite at 4 °C.

bioRxiv preprint doi: https://doi.org/10.1101/2025.02.07.637094; this version posted February 8, 2025. The copyright holder for this preprint (which was not certified by peer review) is the author/funder, who has granted bioRxiv a license to display the preprint in perpetuity. It is made available under aCC-BY 4.0 International license.

Supplemental Figure 3

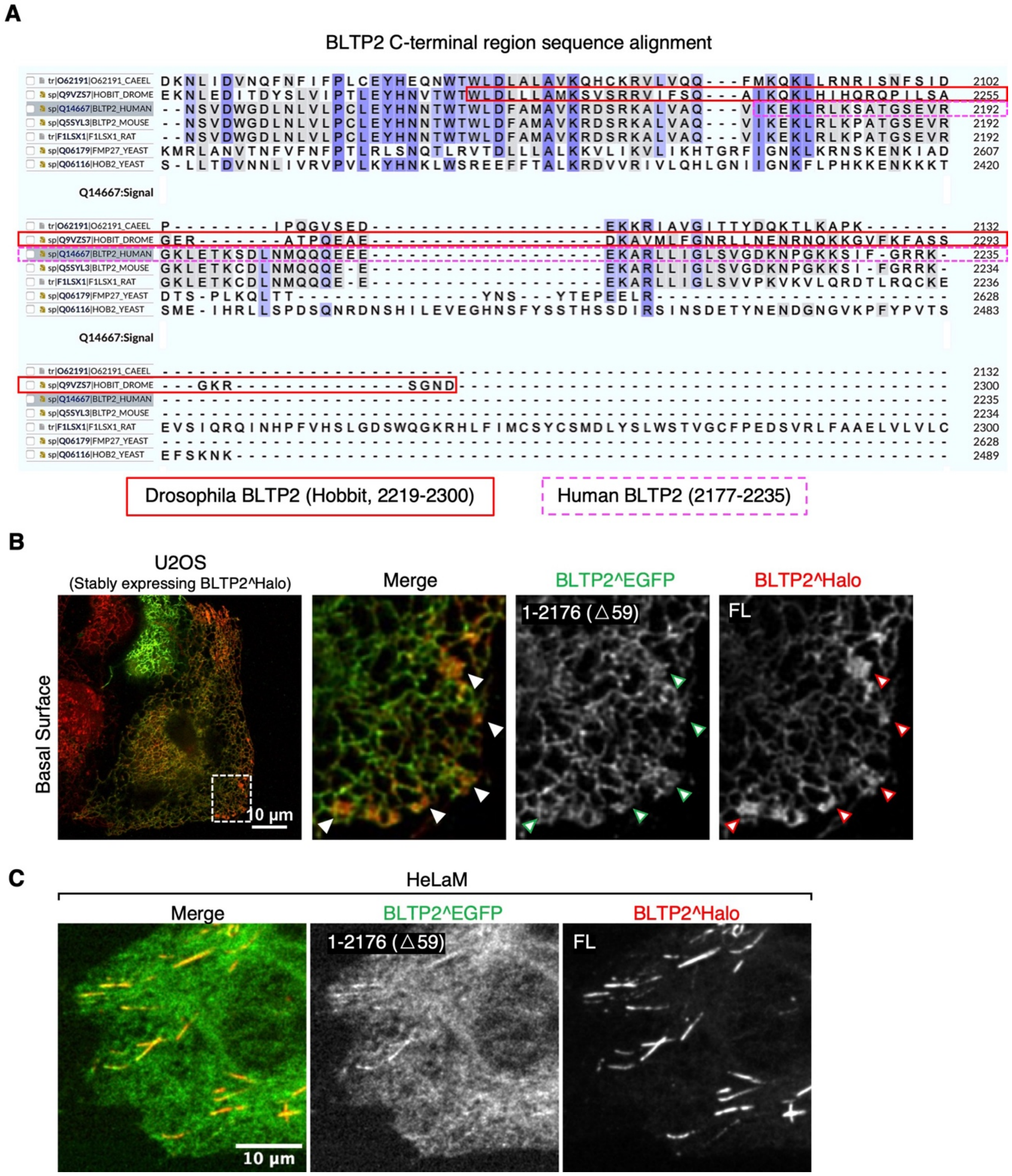

Figure S3. The C-terminal region of BLTP2 is required for its tethering function

(A) Sequence alignment of the C-terminal region of human BLTP2 with the corresponding region of other species. Conserved residues are highlighted in blue, with darker color indicating higher conservation. a.a.2219-2300 of Drosophila BLTP2 (hobbit) are framed by red solid lines and a.a.2177-2235 of human BLTP2 by magenta dashed lines.

(B and C) U2OS cells (B) and HeLaM cells (C) co-expressing the BLTP2 C-terminal deletion mutant BLTP2^EGFP(Δ59) and BLTP2^Halo full length (FL). BLTP2^EGFP(Δ59) show reduced localization relative to BLTP2^Halo FL at ER-PM contact sites in U2OS cells (arrowhead) and at tubular structures in HeLaM cells.

bioRxiv preprint doi: https://doi.org/10.1101/2025.02.07.637094; this version posted February 8, 2025. The copyright holder for this preprint (which was not certified by peer review) is the author/funder, who has granted bioRxiv a license to display the preprint in perpetuity. It is made available under aCC-BY 4.0 International license.

Supplemental Figure 4

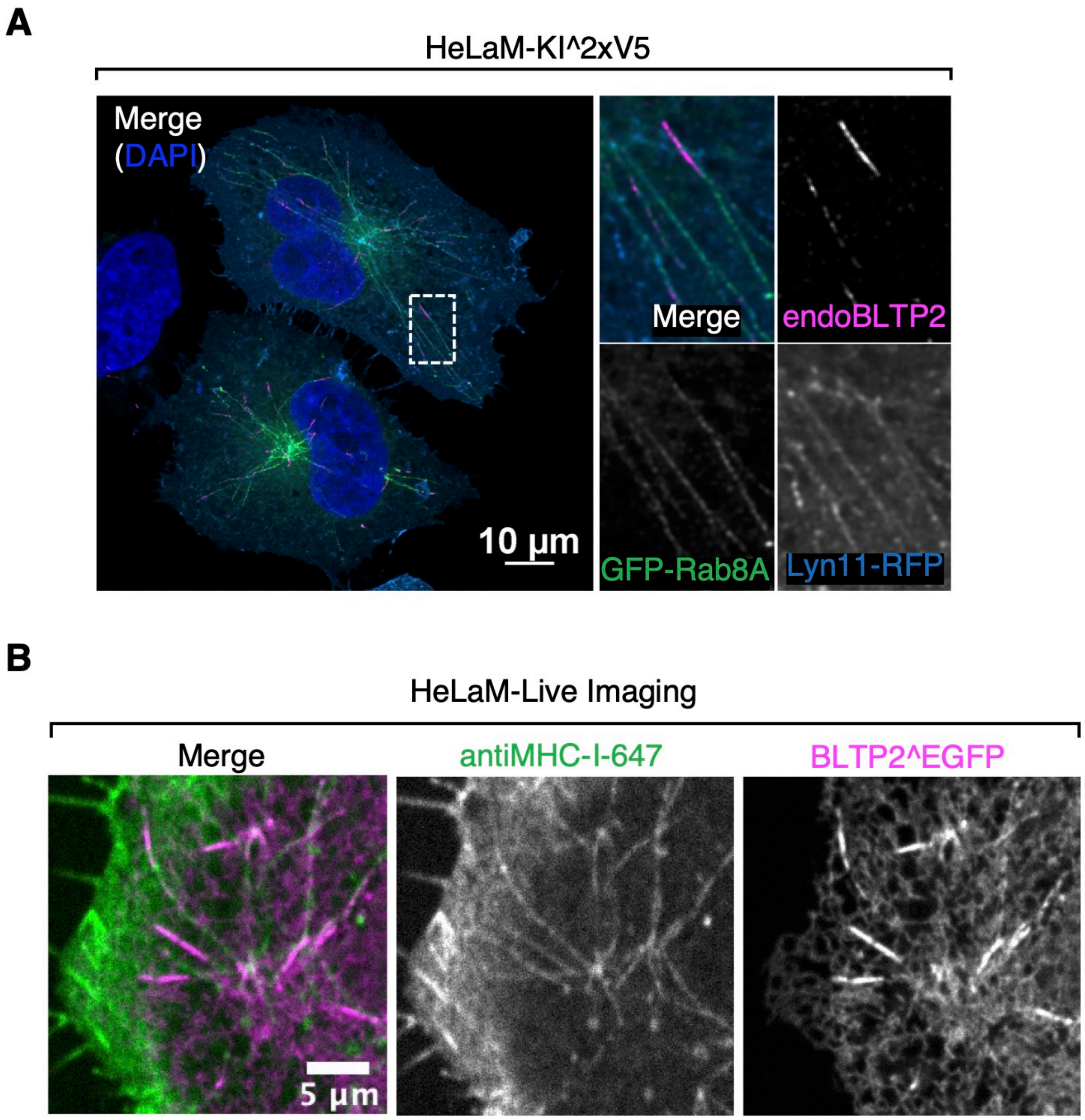

Figure S4. BLTP2-positive tubules have characteristics of tubular recycling endosomes

(A) BLTP2-positive tubules are positive for Rab8A.

(B) A fluorescent labeled MHC-I antibody added to HeLaM cells expression BLTP^EGFP labels the tubules.

bioRxiv preprint doi: https://doi.org/10.1101/2025.02.07.637094; this version posted February 8, 2025. The copyright holder for this preprint (which was not certified by peer review) is the author/funder, who has granted bioRxiv a license to display the preprint in perpetuity. It is made available under aCC-BY 4.0 International license.

Supplemental Figure 5

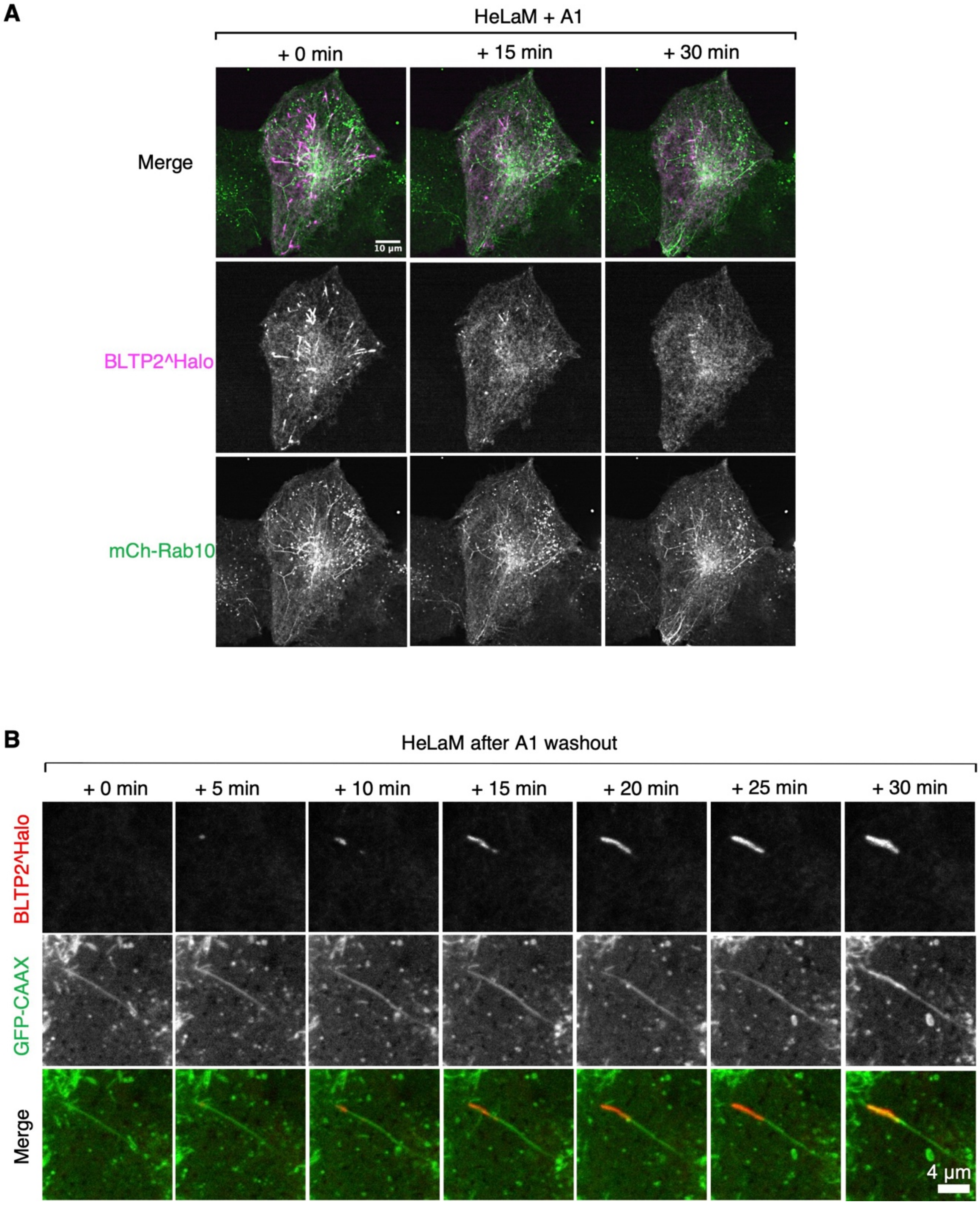

Figure S5. PI4P depletion does not disrupt the Rab10 tubular endosome network

(A) The disassociation of BLTP2 after A1 treatment does not correlate with a disruption of the entire Rab10-positive tubular endosome network in HeLaM cells.

(B) BLTP2 re-establish contact with tubular endosomes after A1 washout.

bioRxiv preprint doi: https://doi.org/10.1101/2025.02.07.637094; this version posted February 8, 2025. The copyright holder for this preprint (which was not certified by peer review) is the author/funder, who has granted bioRxiv a license to display the preprint in perpetuity. It is made available under aCC-BY 4.0 International license.

Supplemental Figure 6

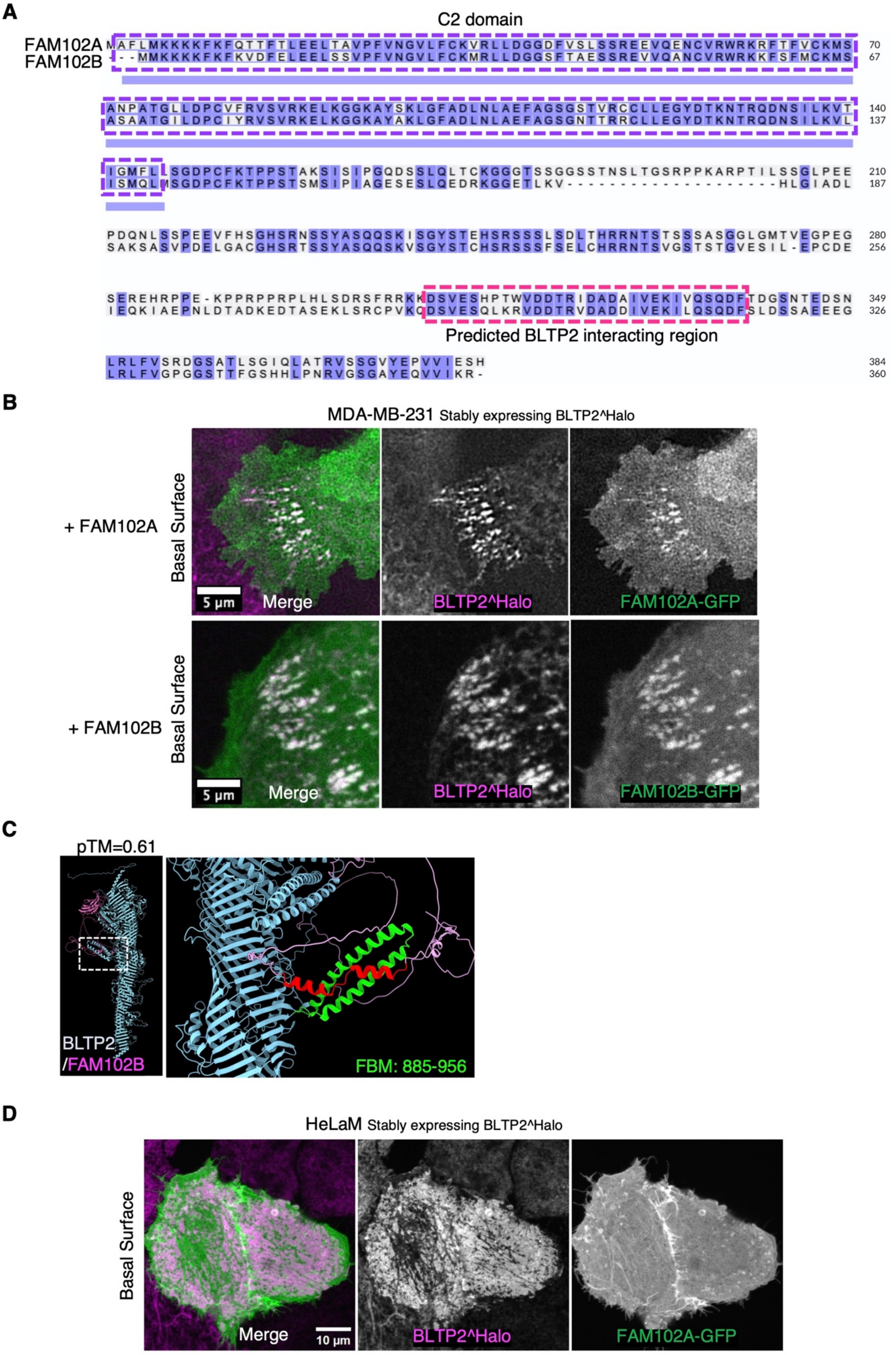

bioRxiv preprint doi: https://doi.org/10.1101/2025.02.07.637094; this version posted February 8, 2025. The copyright holder for this preprint (which was not certified by peer review) is the author/funder, who has granted bioRxiv a license to display the preprint in perpetuity. It is made available under aCC-BY 4.0 International license.

Figure S6. Both FAM102A and FAM102B interact with BLTP2

(A) Sequence alignment of human FAM102A and FAM102B where their N-terminal C2 domains (purple dashed box) and their C-terminal predicted BLTP2 interacting regions (red dashed box) are highlighted.

(B) Co-localization of FAM102A-GFP (Top) and FAM102B-GFP (Bottom) with BLTP2^Halo at ER-PM contact sites in MDA-MB-231 cells.

(C) AlphaFold3 predicted interaction between FAM102B and BLTP2.

(D) FAM102A-GFP recruits BLTP2^Halo to ER-PM contact sites in HeLaM cells.

bioRxiv preprint doi: https://doi.org/10.1101/2025.02.07.637094; this version posted February 8, 2025. The copyright holder for this preprint (which was not certified by peer review) is the author/funder, who has granted bioRxiv a license to display the preprint in perpetuity. It is made available under aCC-BY 4.0 International license.

Supplemental Figure 7

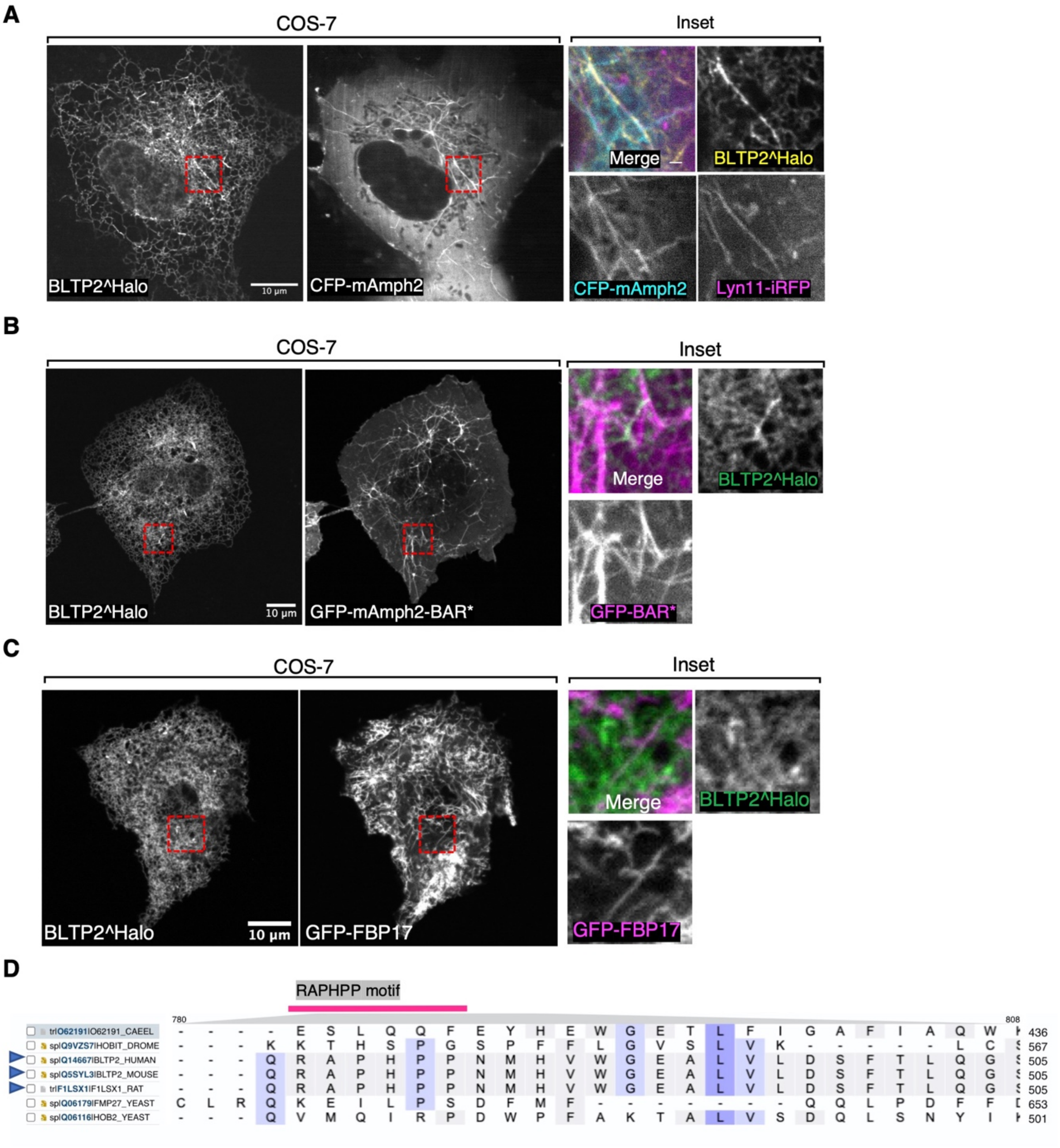

Figure S7. BLTP2 interacts with amphiphysin 2 in an SH3 domain dependent manner

(A) COS-7 cells showing that BLTP2^Halo is recruited to PM tubular invaginations induced by expression of CFP-mAmph2.

(B) COS-7 cells showing that BLTP2^Halo is not recruited to PM tubular invaginations induced by expression of GFP-mAmph2 BAR* which lacks the SH3 domain.

(C) COS-7 cells showing that BLTP2^Halo is not recruited to PM tubular invaginations induced by expression of GFP-FBP17.

(D) Sequence alignment of the region comprising the RAPHPP motif among BLTP2 from several organisms. The RAPHPP motif is conserved among mammalian species (blue arrowhead).

bioRxiv preprint doi: https://doi.org/10.1101/2025.02.07.637094; this version posted February 8, 2025. The copyright holder for this preprint (which was not certified by peer review) is the author/funder, who has granted bioRxiv a license to display the preprint in perpetuity. It is made available under aCC-BY 4.0 International license.

Supplemental Figure 8

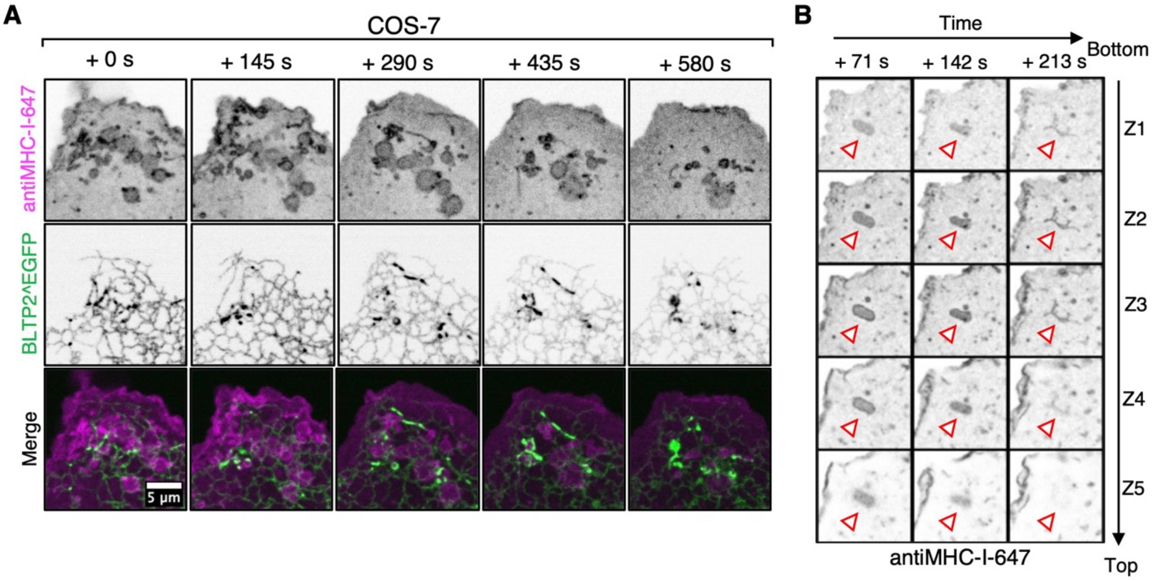

Figure S8. BLTP2 is recruited to macropinosomes undergoing fusion with the PM in COS-7 cells

(A) Acute recruitment of BLTP2^EGFP to macropinosomes (labeled by internalized fluorescent anti MHC-I antibodies) in the process of fusing with the PM.

(B) Z-stack of the time lapse images shown in Figure 6A proving that the macropinosome is not moving out from the focal plane during the imaging session.

bioRxiv preprint doi: https://doi.org/10.1101/2025.02.07.637094; this version posted February 8, 2025. The copyright holder for this preprint (which was not certified by peer review) is the author/funder, who has granted bioRxiv a license to display the preprint in perpetuity. It is made available under aCC-BY 4.0 International license.

Supplemental Figure 9

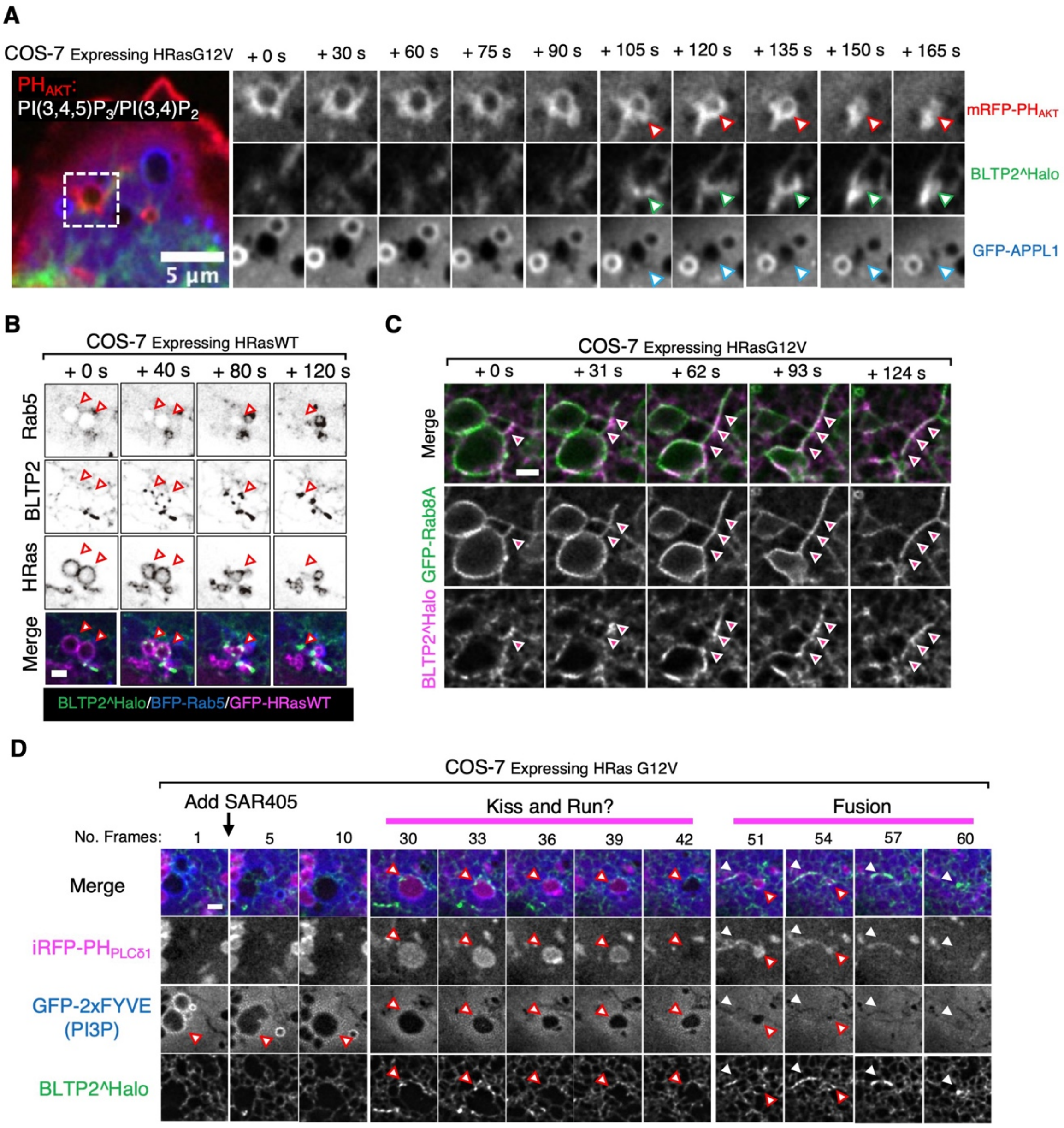

Figure S9. BLTP2-positive recycling macropinosomes are not matured into early endosomal stage

(A) PH_{AKT} also label nascent macropinosomes. The recycling macropinosomes are positive for PH_{AKT} while recruiting BLTP2 and are not positive for APPL1.

(B) BLTP2-positive recycling macropinosomes are not positive for early macropinosome marker Rab5.

(C) BLTP2-positive recycling macropinosomes are positive for exocytic factor Rab8A.

(D) After treating cells with VPS34 inhibitor SAR405, the PI3P positive early macropinosomes can reverse to $PI(4,5)P_2$ and recycle back to the PM.

bioRxiv preprint doi: https://doi.org/10.1101/2025.02.07.637094; this version posted February 8, 2025. The copyright holder for this preprint (which was not certified by peer review) is the author/funder, who has granted bioRxiv a license to display the preprint in perpetuity. It is made available under aCC-BY 4.0 International license.

Supplemental Videos legends

Video S1. HeLaM cell expressing BLTP2^EGFP and mCherry-Rab10 showing the recovery of mCherry-Rab10-positive tubules after the wash out of nocodazole, and the recruitment of BLTP2^EGFP at their tips when they reach the PM.

Video S2. HeLaM cell expressing BLTP2^Halo and mCherry-Rab10 showing dissociation of BLTP2^Halo from mCherry-Rab10-positive tubules after the addition of the A1 compound. The mCherry-Rab10-positive tubule network is not disrupted.

Video S3. HeLaM cell expressing BLTP2^Halo and GFP-CAAX showing recovery of BLTP2^Halo-positive contacts on a GFP-CAAX-positive tubule after the wash out of the A1 compound. The wash-out is carried out after A1 treatment for one hour.

Video S4. COS-7 cell co-expressing BLTP2^Halo (white), FAM102A-GFP (not shown) and M1R-blank (not shown) showing disruption of BLTP2^Halo-dependent ER-PM contacts after the addition of Oxo-M, with the dispersion of BLTP2^Halo throughout the ER and the re-establishment of such contacts after the addition of atropine.

Video S5. COS-7 cell co-expressing BLTP2^Halo (white), FAM102B-GFP (not shown) and M1R-blank (not shown) showing disruption of BLTP2^Halo-dependent ER-PM contacts after the addition of Oxo-M, with the dispersion of BLTP2^Halo throughout the ER and the re-establishment of such contacts after the addition of atropine.

Video S6. COS-7 cell showing a macropinosome labeled by anti-MHC-I-647 antibodies that undergoes a drastic morphological change as it fuses with the PM. This transition correlates with the formation of BLTP2^EGFP positive ER contacts.

Video S7. COS-7 cell showing multiple newly formed macropinosomes labeled by antiMHC-I-647 antibodies that undergo drastic morphological change as they fuse with the PM and acquire patches of BLTP2^EGFP during this transition.

Video S8. COS-7 cell showing a newly formed macropinosome labeled by antiMHC-I-647 antibodies that first loses PI(4,5)P_2 (as detected by GFP-$PH_{PLC\delta1}$) just after internalization, then re-acquires PI(4,5)P_2 as it fuses with the PM. The acquisition of PI(4,5)P_2 (which signals fusion with the PI(4,5)P_2 rich PM) correlates with the formation of BLTP2^Halo positive ER contacts.

Video S9. COS-7 cell expressing HRas G12V and showing a newly formed PI(4,5)P_2-positive (GFP-$PH_{PLC\delta1}$ signal) macropinosome that loses PI(4,5)P_2, but then re-acquires it along with PM dye signal as it fuses back to the PM. BLTP2^Halo is also recruited during its fusion with the PM.

Video S10. BLTP2-KO HeLaM cell expressing HRas G12V showing that a subset of the internally accumulated vacuoles positive for PI(4,5)P_2 (iRFP-$PH_{PLC\delta1}$) become labeled with the

bioRxiv preprint doi: https://doi.org/10.1101/2025.02.07.637094; this version posted February 8, 2025. The copyright holder for this preprint (which was not certified by peer review) is the author/funder, who has granted bioRxiv a license to display the preprint in perpetuity. It is made available under aCC-BY 4.0 International license.

PM dye CellBrite shortly after the addition of this dye, signaling continuity of these vacuoles with the PM.

Video S11. BLTP2-KO HeLaM cell expressing HRas G12V, showing that upon addition to the cells of 10KD-Dextran 488 the lumen of a subset of the internally accumulated vacuoles become positive for this marker within few minutes signaling their accessibility to the extracellular medium.
